# From source to sink: part 2—seasonal dispersion of microplastics discharged in the NW Mediterranean Sea by the Rhone River in southern France

**DOI:** 10.1007/s11356-024-35364-6

**Published:** 2024-10-30

**Authors:** Lisa Weiss, Claude Estournel, Patrick Marsaleix, Guillaume Mikolajczak, Mel Constant, Jean-François Ghiglione, Wolfgang Ludwig

**Affiliations:** 1 Université Toulouse, IRD, CNRS, CNES, UPS, Laboratoire d’Etudes en Géophysique et Océanographie Spatiales (LEGOS), Toulouse, 31400 France; 2 Université de Perpignan Via Domitia, CNRS, Centre de Formation et de Recherche sur les Environnements Méditerranéens (CEFREM), Perpignan, 66000 France; 3 Université Lille, Institut Mines-Télécom, Université Artois, Junia, Laboratoire de Génie Civil et géo-Environnement (LGCgE), Lille, 59000 France; 4 Sorbonne Université, CNRS, Laboratoire d’Océanographie Microbienne (LOMIC), Banyuls-sur-mer, 66650 France

**Keywords:** Plastic debris, River-sea continuum, Lagrangian modeling, Frontal ocean dynamics

## Abstract

**Abstract:**

As the largest individual contributor of freshwater inflow to the basin, the Rhone River is likely to be one of the main sources of microplastics (MPs) to the Mediterranean Sea. In order to predict the fate of MPs discharged by the Rhone River, an innovative 3D Lagrangian dispersion of its particles associated with vertical velocities was modeled in Mediterranean ocean currents. Through winter and summer scenarios, the seasonal variability of transfers and the corresponding accumulation areas were depicted in the Northwestern Basin according to hydrodynamic conditions on the continental shelf of the Gulf of Lion and to the frontal dynamics from the Pyrenees to the North Balearic fronts. Our results indicated that MP transfers were driven by mesoscale and sub-mesoscale structures, resulting in steep concentration gradients across fronts during summer, while winter energetic mixing favored a more efficient and homogeneous spreading. After a year of drift, high MP retention (up to 50%) occurred in the coastal zone of the Gulf of Lion near the river mouth, with a large contribution of sinking MPs and an increase in stranding during the highest freshwater inflows of the winter season. Conversely, up to 60% of the floating MPs were exported to the Algerian Basin and then to the Eastern Mediterranean. This west-to-east transfer led to significant stranding on the islands, prevailing on the northern coasts of the Balearic Islands in winter (6% of floating inputs) and on the western coasts of Corsica and Sardinia in summer (13%). The southern Mediterranean coasts, from Algeria to Tunisia, represented also a major sink for floating debris with stranding ranging from 9 to 35% of MPs discharged in winter and in summer, respectively. We estimated that 3.5 to 5 t of the Rhone MPs remained in the surface layer at the end of the year, with high concentrations in the Ionian Sea.

**Graphical abstract:**

Seasonal distribution of floating and sinking MPs discharged by the Rhone River into the surface and bottom layers of the Mediterranean Sea.
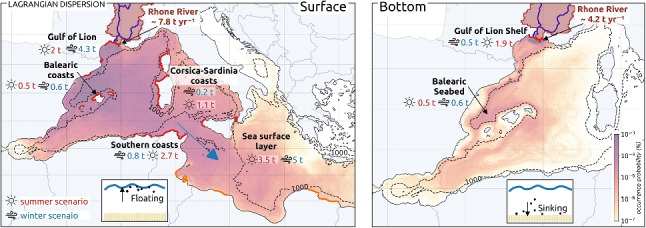

## Introduction

The Mediterranean Sea is designated as a hotspot for plastic pollution since high continental water discharges, high population densities, and intensive tourism exert strong anthropogenic pressure on this semi-enclosed basin (Cózar et al. 2015). Large quantities of mismanaged plastics are trapped and stored on continental surfaces (Schwarz et al. 2023), and rivers are likely the major pathways to efficiently transfer inland-generated plastic debris to the sea (González-Fernández et al. 2021). Rivers therefore play an important role in marine contamination. Most of the plastic (in numbers) entering the sea are in the form of microplastics (MPs) (Strokal et al. 2023), with numerous negative impacts on the organisms (Leistenschneider et al. 2023). Distinction was made between primary MPs, purposefully manufactured in small size (micro-beads used in cosmetics, fibers, industrial pellets), and secondary MPs that result from weathering and breakdown of larger plastic items. Riverbanks are the place of intense transformation of macro debris into secondary MPs during their very slow transfer with transit times up to several decades before reaching the marine environment (Tramoy et al. 2020a, b).

The Rhone, located at the East of the Gulf of Lion, is the most important river in terms of freshwater supply to the Mediterranean Sea, with discharges ranging from 500 to 10,000 $m^{3} \ s^{-1}$, averaged to 1720 $m^{3} \ s^{-1}$ (Ludwig et al. 2009). Other freshwater inflows to the Gulf of Lion come from smaller coastal rivers such as the Tech, Têt, Agly, Aude, Orb, or Hérault rivers, which together contribute to a freshwater discharge much lower than the Rhone (around 140 $m^{3} \ s^{-1}$). Total suspended solids discharged from these rivers constitute on average only less than 6% of the total suspended solids of the Rhone River (Sadaoui et al. 2016). The Rhone River is therefore a significant possible contributor to MP pollution in the Mediterranean Sea. Previous modeling studies on river discharges of MPs to the ocean all highlighted the outstanding role of the Asian, south American, or African rivers as terrestrial sources (Meijer et al. 2021; Lebreton et al. 2017), suggesting that also in the Mediterranean, most MPs are introduced from eastern and southern rivers (Baudena et al. 2022; Soto-Navarro et al. 2020; Liubartseva et al. 2018). However, very recently (Weiss et al. 2021, 2024), this highly heterogeneous view of riverine MP sources has been seriously questioned and the Rhone was indeed identified as one of the major MP pollution hotspots in the Mediterranean (Constant et al. 2020; Castro-Jiménez et al. 2019).

The hydrodynamics on the continental shelf is mainly influenced by two dominant winds, the Mistral at the eastern side and the Tramontane at the western side, by the inputs of the Rhone River and by the Northern Current (Millot 1999) flowing along the continental slope. Further south, thermohaline fronts have a crucial impact on the regional physical oceanography and marine ecosystem productivity (Lévy et al. 2018). The two prominent thermal fronts in the region are the North Balearic Front and the Pyrenees Front (Barral et al. 2021). They mark the interface between the southern warmer less salty waters from the Atlantic and the northern colder saltier Mediterranean waters, forming convergence zones and influencing the distribution of nutrients, phytoplankton, marine organisms (Olita et al. 2014) but also floating pollutants as plastic debris (D’Asaro et al. 2018; Cózar et al. 2021). Investigating the role of thermal fronts and associated sub-mesoscale structures in the dispersion and retention of MPs is therefore crucial to scale future monitoring and mitigation efforts and to assess potential risks to marine ecosystems. At the river mouths, denser MPs may also be exported and transported vertically into the water column to be submitted to a combination of sinking rates and deep currents (Weiss et al. 2024).

Our objective was to analyze the seasonal dispersion of MPs exported by the Rhone River exploring a summer and a winter scenario. It was partly based on a more general modeling experiment which used the current fields calculated by the ocean model SYMPHONIE (Marsaleix et al. 2006, 2008) over the Mediterranean basin and its Lagrangian tracking module which was modified and adapted to MP transport (Weiss et al. 2024). Contrary to this previous simulation framework, we introduced two seasonal scenarios, which gave a better understanding of the impact of currents on MP dispersion in the Gulf of Lion and the Northwestern Mediterranean frontal zone. Moreover, it further allowed to identify and quantify the potential sink areas of MP pollution discharged by the Rhone River, which is an important issue in advocating clean-up and ecosystem conservation actions and in raising public awareness about individual and collective responsibility for the plastic pollution problem.

## Method

### Rhone river MPs

#### MP fluxes

Following the method described by Weiss et al. (2024), the modeled mass fluxes of the Rhone of about 12 $t \ yr^{-1}$ (Weiss et al. 2021) were converted into fluxes in number of MPs using the conversion factors equal to 0.745 $\mu g$ for fibers and 0.233 *mg* for non-fiber MPs (based on literature samples analyzed and illustrated in the supplementary material of Weiss et al. (2021), those factors are associated with uncertainties that do not capture the strong heterogeneity of MPs in marine environment). The resulting flux was about 102.27 $10^{9}$ MPs $yr^{-1}$ (i.e., 280,192 $10^{3}$ MPs $day^{-1}$), considering 50% of synthetic fibers and 50% of non-fiber MPs. These estimates are associated with significant uncertainties due to the lack of extensive in situ observations, the variability of sampling and analytical methods, and the complex and diverse nature of MPs in the environment. However, seasonal variations in MP fluxes of the same order of magnitude—ranging from 5.92 $t yr^{-1}$ during calm periods to 22 $t yr^{-1}$ during floods—were observed by Constant et al. (2020). Thus, the previous assumptions should still be interpreted with caution, as they may lead to potential spatio-temporal quantitative uncertainties.

**Fig. 1:**
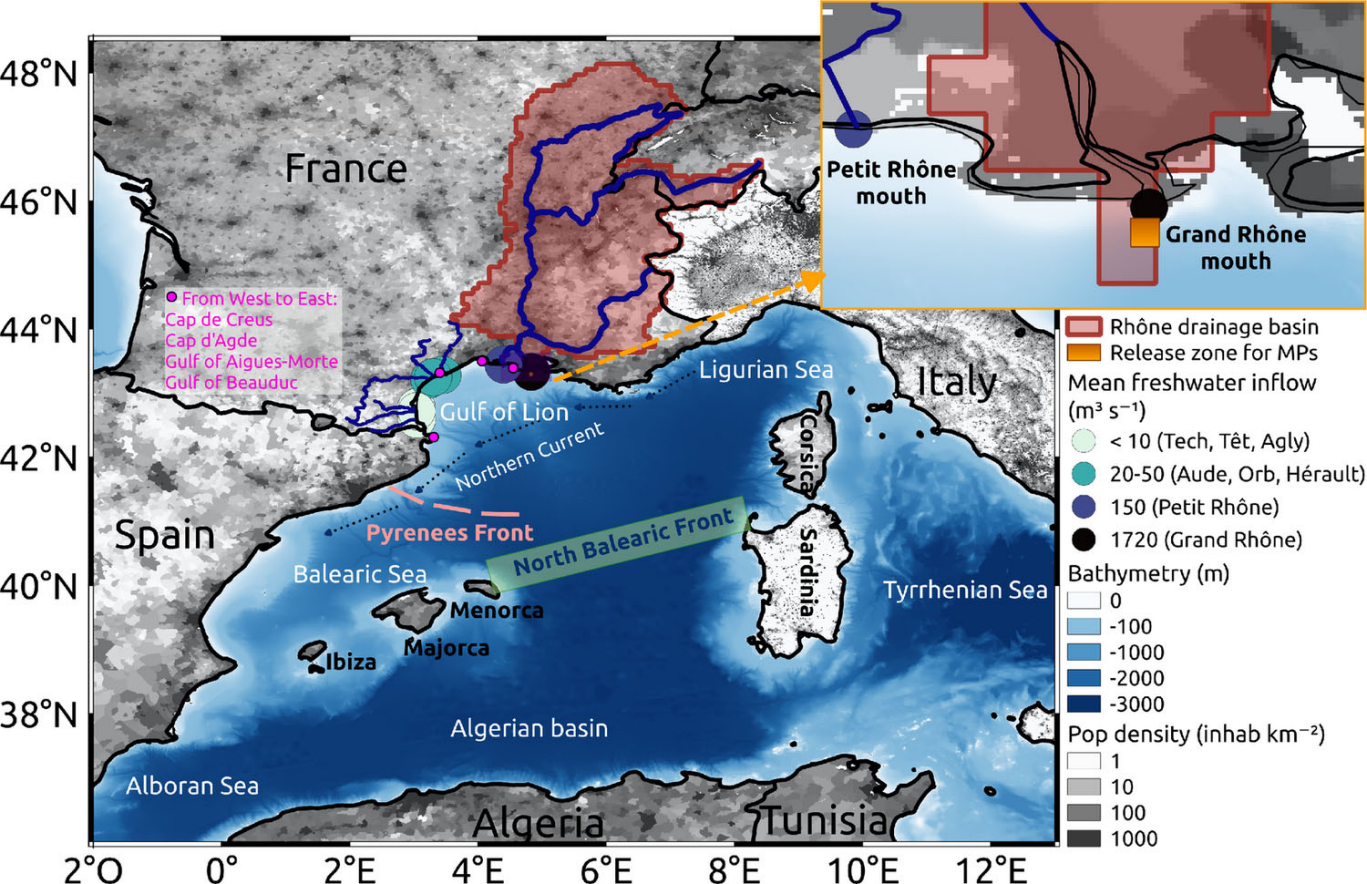
Regional map of the Western Mediterranean Sea with the Rhone drainage basin (at 30 arc-second resolution), its main mouth (zoom), and the 0.04$^{\circ }$ MP release area (in orange)

To overcome the limited number of virtual particles released in the model because of calculation constraints, we deployed one virtual particle counting for $10^{3}$ MPs, leading to a daily release of 280,192 particles $day^{-1}$. The Rhone splits into two channels 50 km north of the coastline, but only its larger easternmost channel was considered in this paper, designated as the Grand Rhone (Fig. 1). The MP release positions were randomly drawn within a 0.04$^{\circ }$ square area delineated off the Grand Rhone River mouth (Fig. 1), representing about 4.5 km on a meridian and 3 km on a parallel at the latitude 43$^{\circ }$.

#### Seasonal scenarios

To study the seasonal dispersion of the MP plume, two numerical experiments were tested, one starting in winter and the other in summer. The winter scenario was based on a continuous release of MPs over the month of February, the coldest month of the year with strong vertical mixing and river discharge. The summer scenario was based on a continuous MP release over August, the warmest month of the year with sea stratification and low river discharge. A total of 8,685,952 $10^{3}$ MPs were released in both scenarios at a rate of 280,192 $10^{3}$ MPs $day^{-1}$. Within a day, the release time of each particle was determined by randomly drawing the hour, minute, and second. Our scenarios do not include a spin-up period for MP concentrations at sea, i.e., they start from a pollution-free state. These scenarios are therefore not intended to represent a realistic quantitative distribution resulting from long-term accumulation, but rather to allow us to specifically analyze the dispersion of two plumes during two seasons with different ocean circulation dynamics. Those 1-month numerical releases at sea in front of the Grand Rhone mouth were followed by a full year of advection of these river MPs into ocean currents to analyze their trajectories.

#### MP vertical motion

Specific rising and sinking vertical velocities associated with the riverine MPs were calculated according to observed sizes in river samples (0.3 to 5 mm), polymer types (with densities between 10 and 1600 $kg \ m^{-3}$), and shapes (fibers, fragments, beads, foams) distributions, as this has been described in detail by Weiss et al. (2024). This resulted in simulated flux of 65% floating MPs, i.e., 182,125 $10^{3}$ MPs $day^{-1}$, and of 35% sinking MPs, i.e., 98,067 $10^{3}$ MPs $day^{-1}$. The rising velocities ranged between [$10^{-2}$, 230] $mm \ s^{-1}$ and the sinking velocities between [-170, -$10^{-2}$] $mm \ s^{-1}$.

**Fig. 2:**
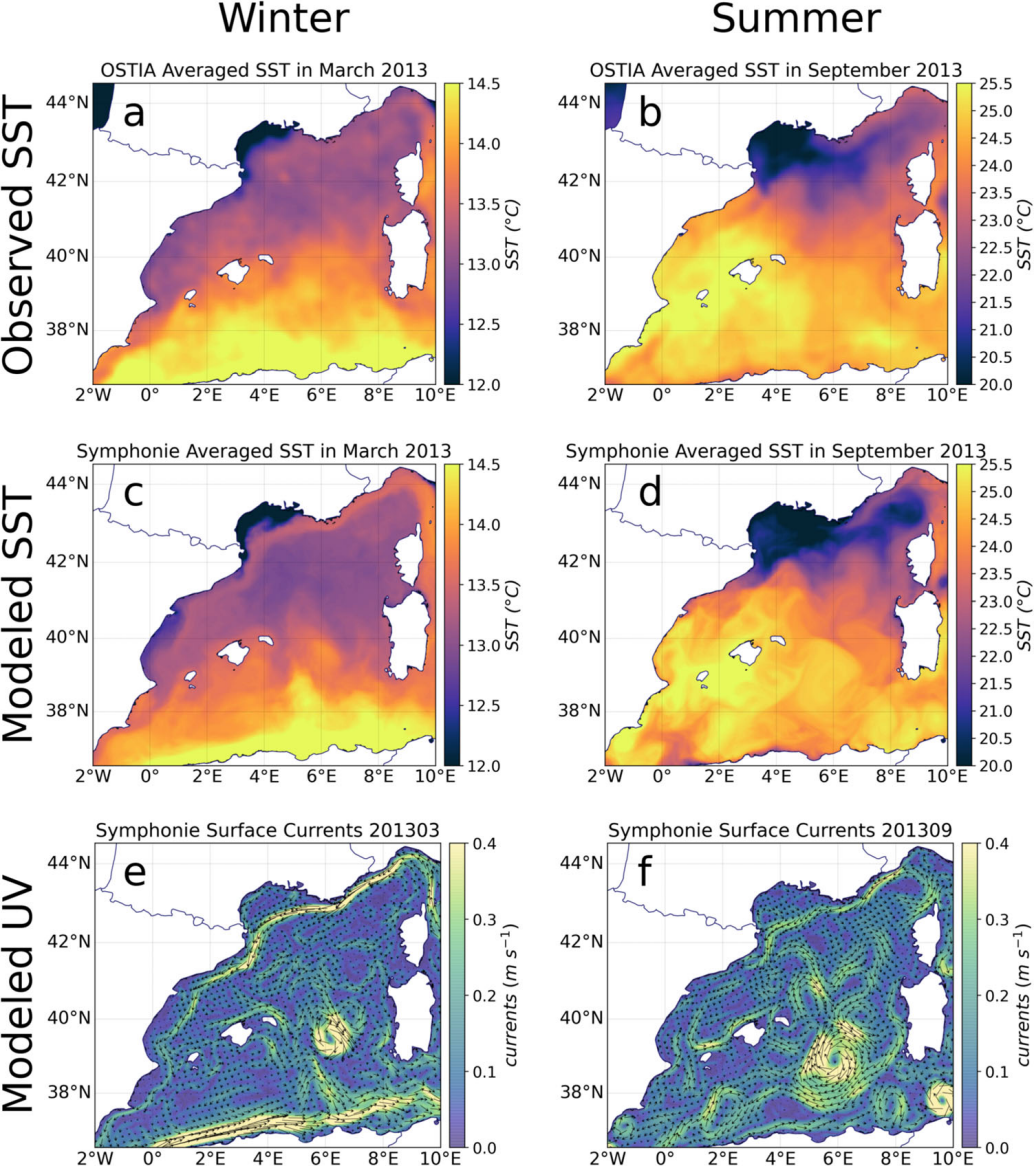
Ocean dynamics in the NW Mediterranean Sea in March and September 2013, according to **a**, **b** the monthly averaged OSTIA SST (0.05$^{\circ }$ horizontal resolution based on *in situ* and satellite data); **c**, **d** the SYMPHONIE modeled SST; and **e**, **f** the SYMPHONIE averaged surface currents

### Lagrangian tracking

#### Description of the ocean simulation

The ocean simulation exploited in our study was performed with the three-dimensional hydrodynamic model SYMPHONIE (Marsaleix et al. 2006, 2008). This model solves the primitive equations for mass and momentum conservation and is based on Boussinesq approximations and hydrostatic equilibrium. The model, the grid, and the forcings used were detailed in previous studies (Estournel et al. 2021; Weiss et al. 2024). The horizontal resolution was about 2–3 km in the Northwestern Mediterranean with 60 VQS (vanishing quasi-sigma) vertical levels. Daily realistic freshwater discharges were simulated at the mouth of the Rhone River. The SYMPHONIE simulation of ocean currents used in this study has been performed over the entire Mediterranean Sea for 2013–2014 (available through the INSU SIROCCO service, sirocco.obs-mip.fr) and validated by Estournel et al. (2021). Through our analysis, 2013 appeared to be representative of usual seasonal variability in the frontal region (see Fig. 2).

It should be noted that one difference with the study by Weiss et al. (2024) is that the simulation used here is not forced by Stokes drift. The omission of the current-wave interaction through Stokes drift in this study may be considered as a potential limitation, particularly in the interpretation of the dispersion results for floating MPs (surface currents are the most affected by Stokes drift, with Stokes velocities ranging from 0.05 to 0.2 $m \ s^{-1}$, whose influence decreases significantly with depth) and during winter (when the intensity of wind events is higher). By taking Stokes drift into account here, we could have expected a potentially increased export of floating MPs in a southerly direction and even a potentially reduction in the stranding of MPs on the Gulf of Lion coast during winter.

#### MP trajectory calculation

Using this previously described SYMPHONIE physical simulation, Lagrangian trajectories were calculated offline using the 3D daily averaged current fields (advection and diffusion terms) for 2013–2014. The SYMPHONIE Lagrangian module includes the general equations for Lagrangian integration (with a time step of 780 s), the two-stage second-order Runge–Kutta advection scheme, the random walk turbulent vertical diffusion scheme, and the semi-empirical rising and sinking velocity calculation, as previously described (Weiss et al. 2024).

#### Sink configuration

MP sequestration in the sediment was not parameterized in our configuration. MPs cannot settle on the bottom and were kept in the first water grid cell above the seafloor where currents were usually very slow. When particles reached the coast, i.e., the edge of the last water grid cells where currents were very weak, they could remain motionless for very long periods. They were considered definitively stranded when they moved less than 100 m in 1 month of simulation. When particles reached the open boundaries, i.e., exit the Strait of Gibraltar, they disappeared from the simulation.

#### Mapping

**Fig. 3:**
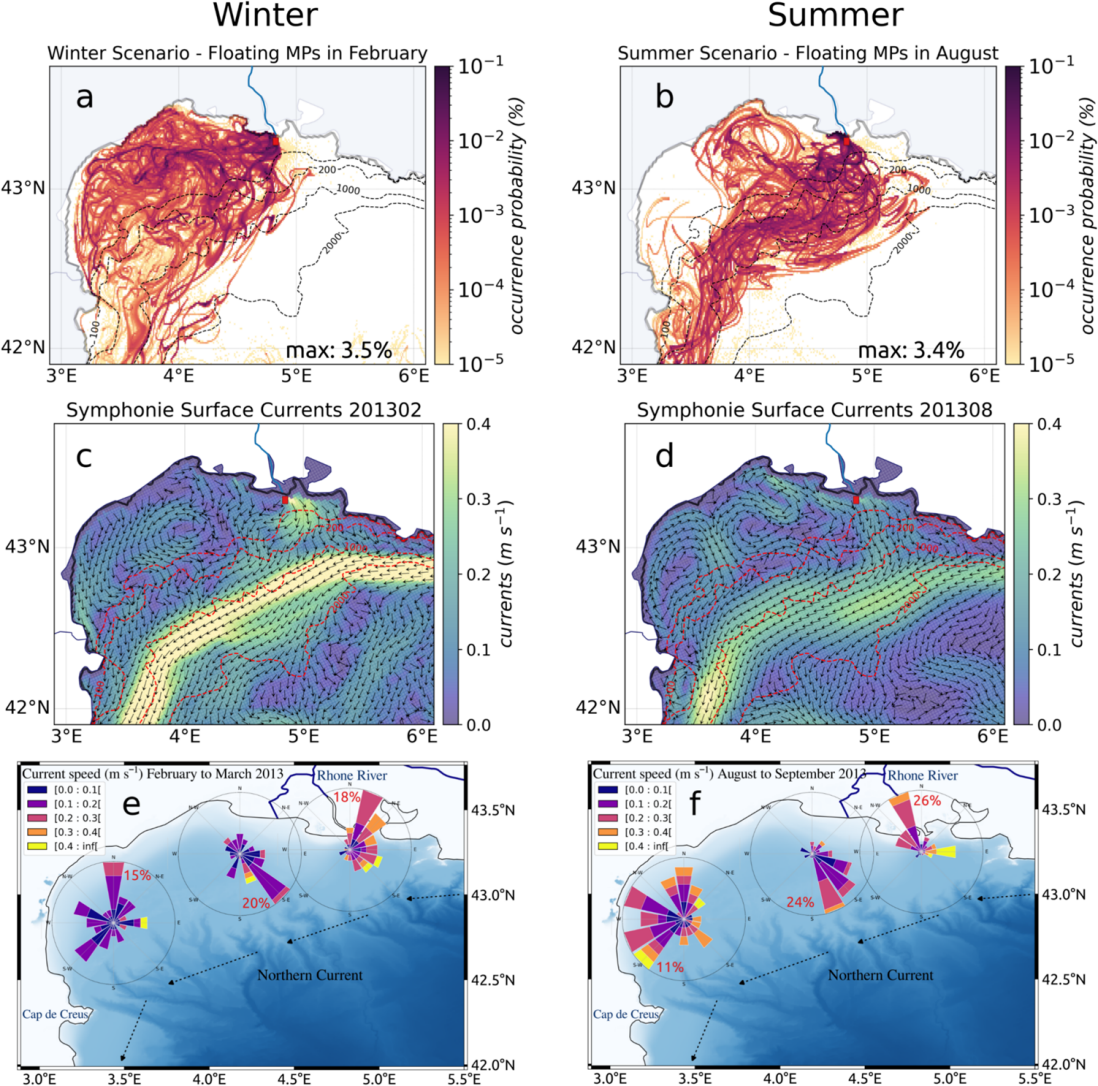
**a**, **b** Occurrence probability maps for floating MPs discharged by the Rhone in February for winter and in August for summer; **c**, **d** corresponding surface currents averaged over these same months; and **e**, **f** surface current roses at three locations. The colors constituting the roses indicate the current speed and the arrow size indicate the frequency of each current direction

A common way to highlight the preferential trajectories of MPs was to represent occurrence probability maps (for example, see Fig. 3a, b). First, density maps were plotted by recording the MP positions every 2 days during the whole simulation (1 year for the two scenarios) and by binning the total number of positions in each grid cell to form 2D histograms (0.01$^{\circ }$ resolution in the following figures). These density histograms were normalized by the total number of MP positions in the simulation to provide occurrence probabilities. The sum of all grid cell probabilities was equal to 100%.

## Results and discussion

### Hydrodynamic context

#### The Northern current

The Northern Current is the most stable current in the Northwestern Mediterranean and corresponds to the northern branch of the cyclonic gyre circulation of the basin (Millot 1999). It flows westwards along the continental slope from the Ligurian Sea to the Balearic Sea (Figs. 1 and 2e, f), with seasonal variations in current amplitude and thickness (Guihou et al. 2013). Depending on the stratification and other processes still poorly understood, a variable part of this current exits south towards the Algerian Basin through the Ibiza channel, while another part flows northeastward along the Balearic Islands (Pinot et al. 2002) and then turns southward around Menorca toward the Algerian basin. The southward path is strengthened in winter (Fig. 2e), while the northeastward branch is dominant in summer (Fig. 2f), in agreement with the observations of Pinot et al. (2002).

#### Frontal zone definition

The regional comparison of monthly averaged SST modeled by SYMPHONIE (Fig. 2c, d) with satellite observations (Fig. 2a, b) from OSTIA (Good et al. 2020) showed good agreement for both seasons distinguished in our study. These SST maps highlighted the main surface currents and seasonal thermal fronts. The nature and position of the western Mediterranean fronts are subject of divergence in the literature. Using a gradient-based method on a 20-year reanalysis, Barral et al. (2021) showed that, as depicted in our simulation, two frontal zones may co-exist in this region with a thermal variability driven by seasonal cycles.

First, the North Balearic Front is a salinity front matching with a thermal front during winter and spring. It extends southeastwards from Menorca to Sardinia which defines the northern limit of the warmer and fresher Atlantic Water spreading into the Algerian basin and the southern limit of the modified Atlantic Waters of the Liguro-Provençal basin, cooled by the strong Mistral and Tramontane winds (Estournel et al. 2016; Herrmann et al. 2017; Testor et al. 2005). This front forms the southern branch of the cyclonic gyre toward the West Corsica Current (Millot 1999; Garreau et al. 2018; Seyfried et al. 2019). In March 2013, the North Balearic Front was diffused both in the satellite image and in the simulation (Fig. 2a, c).

The Pyrenees-Corsica Front is a thermal front extending northeastward from the Catalan coast south of Cap de Creus to Corsica (around 3$^{\circ }$ E and 42$^{\circ }$ N, Figs. 1 and 2b, d) and is present only in late summer and autumn. Its western section is referred to as the Pyrenees Front, already distinguished by Pascual et al. (2002). It separates the warm surface waters of the Balearic Sea from the cold waters of the Gulf of Lion. This front was clearly depicted by our simulation in September 2013 (Fig. 2d), and we can observe an eastward recirculation of the Northern Current following it in late summer (Fig. 2f). From observations, altimetry, and modeling, Carret et al. (2019) noticed that this situation can extend to autumn with a significant inter-annual variability. This branch, exhibiting meanders and eddies, closes the cyclonic circulation of the sub-basin much further north than in winter, partially bypassing the Balearic Sea (Fig. 2f). Moreover, both the satellite images and our simulation indicated an extension of cold water to the southwest along the Catalan coast, separating the two opposite shores of the Balearic Sea by a thermal front (Fig. 2b, d).

South of the North Balearic Front, between the Balearic Islands and Sardinia, a strong anticyclonic eddy of varying size and position was modeled (Fig. 2e, f). Further southwest, it can be noted that the Algerian Current did not develop enough mesoscale instabilities in winter in the simulation (Fig. 2c) when compared to satellite observations (Fig. 2a).

Finally, the dynamic of the year 2013 exhibited significant seasonal variability driven by temperature gradients with possible positions of the frontal zones between 40$^{\circ }$ N in winter and 41.5$^{\circ }$ N in late summer (Fig. 2). The fact that it has already been observed indicates that the following analysis for the year 2013 can be considered as quite representative of usual seasonal variability in the region, even if the fronts position can shift by about 1$^{\circ }$ between years, depending on the intensity of deep-water formations in the Liguro-Provençal basin and the instability of Algerian eddies (Barral et al. 2021).

### Dispersion of MPs in the Gulf of Lion

#### Floating MPs in the surface layer

In both summer and winter, MPs exited the continental shelf via the southwestern part of the Gulf (Fig. 3a, b), following the Northern Current (Fig. 3c, d). However, a striking difference was observed on the western part of the shelf. In winter, the circulation was mainly cyclonic, leading to a widespread distribution of MPs across the entire shelf. Whereas in summer, an anticyclonic cell, previously noticed by Millot (1982) and Hu et al. (2009), deflected the floating MPs and prevented them from getting close to the western coasts, from the french Cap d’Agde to the Cap de Creus in Spain, where is located the Marine Protected Area of the Gulf of Lion, thus drastically decreasing the risk of contamination and stranding on these coasts at this period. The high occurrence probabilities at 43$^{\circ }$ N, 4$^{\circ }$ E in winter could be explained by a second cyclonic cell conducive to MP accumulation. In both seasons, the most affected coasts were those to the north, from the western limit of Gulf of Aigues-Morte (marked by the Palavas-les-flots harbor breakwater) to the Cap d’Agde. Interestingly, the Gulf of Aigues-Morte was protected in winter but became contaminated in summer. The opposite pattern was observed in the Gulf of Beauduc to the southeast, which is adjacent to the Camargue Regional Nature Park and experienced higher contamination in winter.

**Fig. 4:**
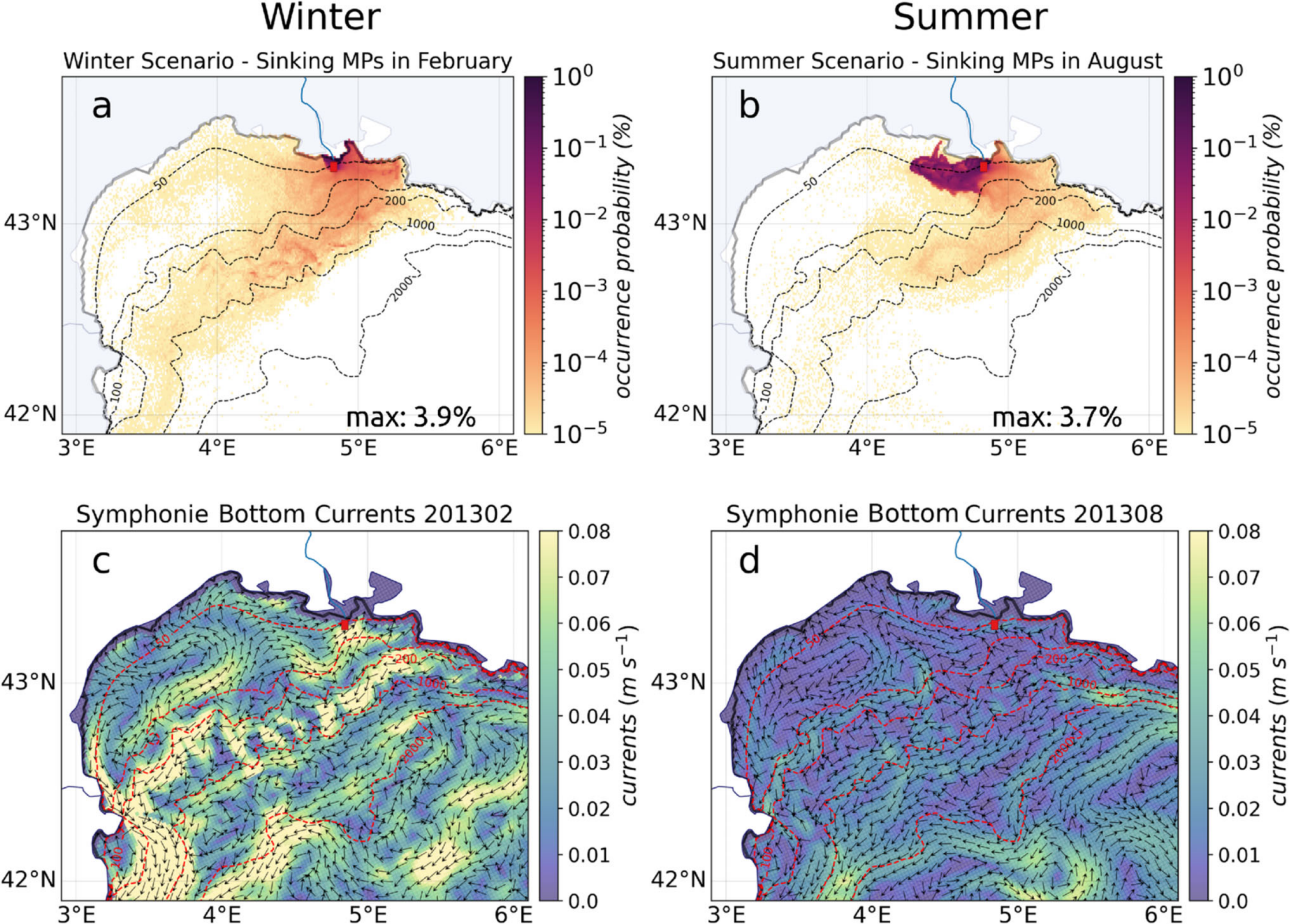
**a**, **b** Occurrence probabilities for sinking MPs in the Gulf of Lion in February for winter and in August for summer and **c**, **d** corresponding averaged bottom currents

A more detailed analysis of the Lagrangian trajectories simulated was difficult due to the chaotic nature of the shelf dynamics. However, our results align with in situ observations (Constant et al. 2020; Castro-Jiménez et al. 2019; Schmidt et al. 2018), highlighting the important role of the Rhone River in the floating MP dispersion, especially during winter. The Rhone freshwater plume, supplied by higher discharge during this season, interacts with the cyclonic circulation observed on the shelf (Estournel et al. 1997, 2003) and contributes to the wide distribution of MPs, affecting more sensitive and protected areas across the shelf. In contrast, the reduced Rhone discharge in summer further attenuates an already less extensive dispersion. Moreover, Constant et al. (2020) showed that Rhone flooding, associated with winter storms, significantly increases MP fluxes towards the sea (from 5.92 $t yr^{-1}$ to 22 $t yr^{-1}$), exacerbating their dispersion pattern during this season. These winter processes accentuate current variability, making the floating MP dispersion more chaotic and less predictable.

In order to illustrate the high variability of surface wind-driven currents in this region subject to strong winds, Fig. 3e and f shows surface current roses in late winter and late summer at three locations in the Gulf of Lion. Although some current directions, corresponding to the prevailing wind induced circulation, were dominant at each location, we observed that the direction of current fields could be highly variable with associated high current intensities. For example, at the Rhone River mouth, the majority of the currents came from the North, corresponding to the direction of the Rhone plume during the prevailing northerly winds (Estournel et al. 1997). However, high-speed currents flowing from the east corresponding to eastern storms (Estournel et al. 1997; Gangloff et al. 2017) also affected the area, being responsible for the wide dispersion of MP trajectories toward the inner shelf.

**Fig. 5:**
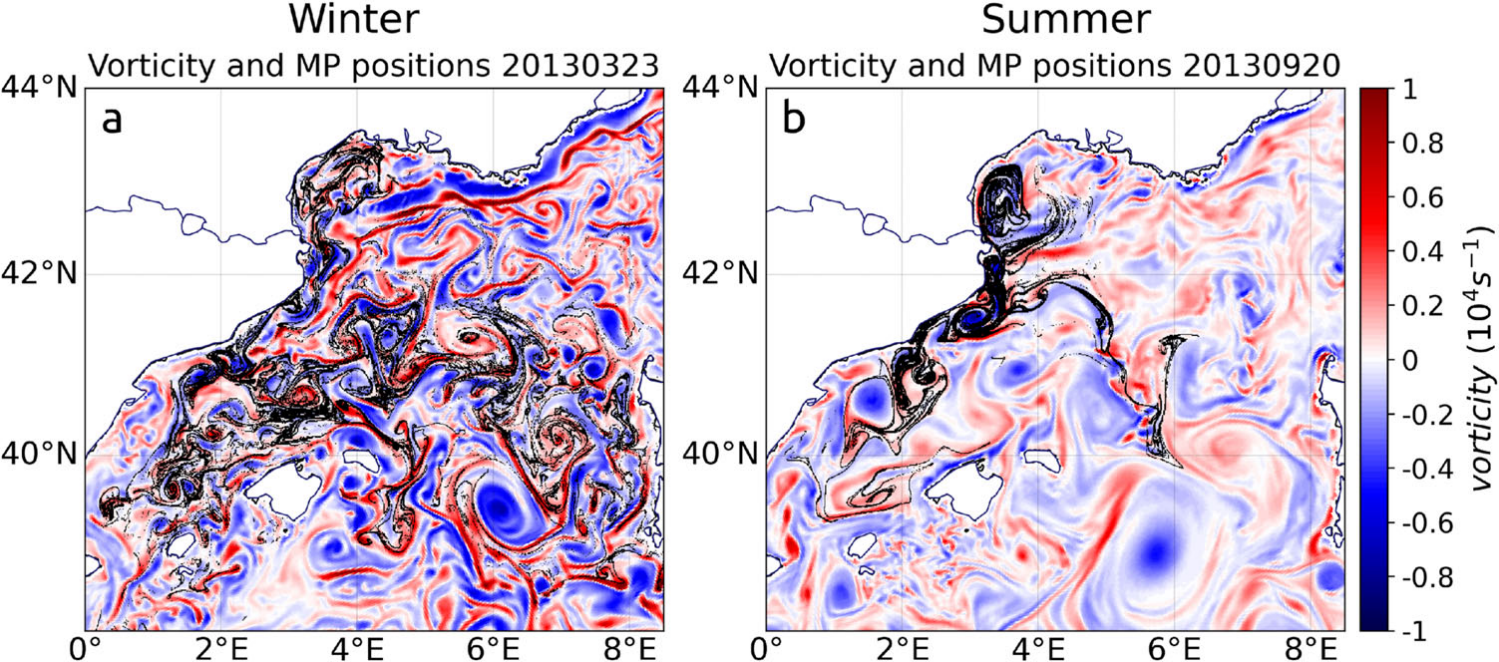
Snapshots of floating MP positions superimposed to the relative vorticity calculated from SYMPHONIE current fields (anticyclonic in blue and cyclonic in red) 3 weeks after the last release **a** in winter (23 March 2013) and **b** in summer (20 September 2013)

#### Sinking MPs at depth

The current speed near the seafloor was up to three times lower than at the surface (Fig. 4c, d). Export of sinking MPs out of the continental shelf was therefore limited. In both seasons, a large amount of sinking MPs stayed at shallow depths during the whole 1-year simulation, 86–88% between the coast and 20 m. Thus, the mean depth of sinking MPs including stranded MPs remained around 20 m. This reflects what has been observed in other deltaic environments, such as the Po prodelta, where strong accumulation of MPs in sediments has been documented (Pellegrini et al. 2023). In our results, two clusters of sinking MPs could be distinguished in both seasons (Fig. 4a, b). The first represented the densest MPs with higher sinking velocities with a median at -17 $mm \ s^{-1}$ ( in [-170, -5] $mm \ s^{-1}$). These sinking velocities are one to two orders of magnitude higher than the vertical velocities of the currents (of the order of ± 0.01 to 1 $mm \ s^{-1}$), causing these MPs to reach the bottom very quickly and very close to the river mouth (higher occurrence probabilities in Fig. 4a, b). The other cluster was more dispersed (lower occurrence probabilities) and represented MPs with lower sinking velocities with a median at $-$2.7 $mm \ s^{-1}$ (in [-5, $-$0.01] $mm \ s^{-1}$, mainly fibers or $<1 \ mm$ light beads and fragments), which are therefore much more influenced by vertical current fields and could travel greater distances.

The bottom currents were stronger in winter than in summer (a ratio of about 4, Fig. 4c, d) due to the summer stratification which restricted the penetration of the momentum input from the wind (itself also weaker in this season). In the vicinity of the Rhone mouth, the sinking MPs released in summer were mainly transported westward (Fig. 4b), while those released in winter remained trapped in front of the river or wash ashore directly near the mouth (Fig. 4a). In winter, the freshwater inflow of the Rhone was stronger (Fig. 3c), leading to a counter-current of salty water directed towards the river mouth below the freshwater plume (Fig. 4c). On the contrary, during the low freshwater inflow summer period, this bottom current no longer existed or was weak and restricted to the salt wedge inside the Rhone (as described by Thill et al. 2001). Instead, a weak coastal current flowed westward corresponding to the residual circulation of the Northern Current (Fig. 4d), redirecting massively sinking MPs towards the west of the shelf (Fig. 4b). This situation was reflected by a massive exit of the MPs outside the 10 km coastal zone in August (98% MPs illustrated Fig. 8, until day 31 in summer) with a decrease in the mean MP depth to 70 m.

In agreement with previous observations of Alberola et al. (1995); Birol et al. (2010); Carret et al. (2019) and Sammari et al. (1999), the Northern Current along slope was deeper and stronger in winter than in summer (Fig. 4cd), leading to a more rapid export off the shelf of sinking MPs that were not stranded yet or trapped near the mouth. As it happened at the surface, there was an inflow of MPs to the west of the shelf in winter during eastern storms, whereas in summer, this area was still naturally sheltered (Fig. 4a, b). This pattern may be related to the important role of the Rhone River in exporting sediments to the canyons and deep sea, especially during winter with frequent strong winds (Mikolajczak et al. 2020; Estournel et al. 2023). In addition to the sinking MPs carried by the Rhone freshwater discharge, the river sediments themselves contain high concentrations of MPs and associated additives (Vidal et al. 2024). Huge amount of marine litter have been observed in these canyons and on the continental shelf (Tubau et al. 2015; Galgani et al. 2000; Gerigny et al. 2019).

### Dispersion in the western basin

In contrast to the shelf, where currents exhibited high temporal variability over short periods, there was a prevailing seasonal variability that imprinted the distribution of MPs in the western basin, closely related to the main thermal and haline fronts (Fig. 2).

**Fig. 6:**
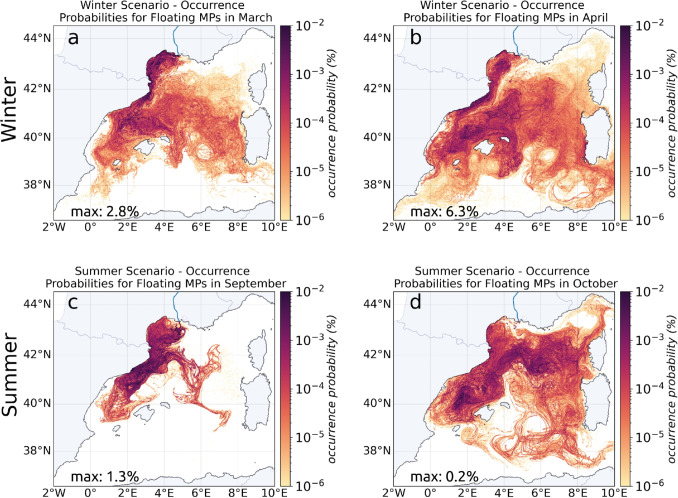
**a**, **b** Occurrence probabilities in late winter to spring (March–April) for MPs released in February and **c**, **d** monthly occurrence probabilities in late summer to autumn (September–October) for MPs released in August

#### Role of the sub-mesoscale

A wide range of scales drive the circulation in the Northwestern Mediterranean Sea, from basin scale currents (i.e., Northern Current, West Corsica Current) associated with persistent fronts to mesoscale motions with a Rossby deformation radius of the order of a few kilometers in winter, providing the straining and stirring environment favorable to the emergence of short-lived fronts. Both persistent and short-lived fronts are associated with sub-mesoscale dynamics, in particular ageostrophic secondary circulation, which induces surface convergence of floating debris (D’Asaro et al. 2018; Cózar et al. 2021), filaments, and sub-mesoscale vortices (Bosse et al. 2021; Damien et al. 2017). Persistent fronts such as the North Balearic Front can also develop negative potential vorticity especially forced by strong heat losses and down-front winds triggering sub-mesoscale symmetric instabilities testifying of important frontal vertical movement (Seyfried et al. 2019; Bosse et al. 2021). Sub-mesoscale short-lived fronts were much more energetic in winter than in summer as the mixed layer depth regulates the amount of potential energy available to generate kinetic energy (Gula et al. 2014). They laterally extended on a few kilometers only (1–10 km) and rapidly evolved.

Comparing the instantaneous floating MP positions bet-ween winter and summer with the corresponding vorticity fields (Fig. 5) showed the effect of the sub-mesoscale on their dispersion. The positive buoyancy of those floating MPs played a key role in the barrier and retention effects of fronts. Indeed, the convergence of surface water flows lead to a downwelling in the middle of the filaments with instantaneous vertical velocities from 0.01 to 1 $cm \ s^{-1}$ (Bosse et al. 2021; Gula et al. 2014; Vic et al. 2022). If the MPs were passive (0 vertical velocities), they would therefore be dragged in depth, whereas here, floating MPs, with rising velocities, tended to concentrate in such surface convergence zones.

**Fig. 7:**
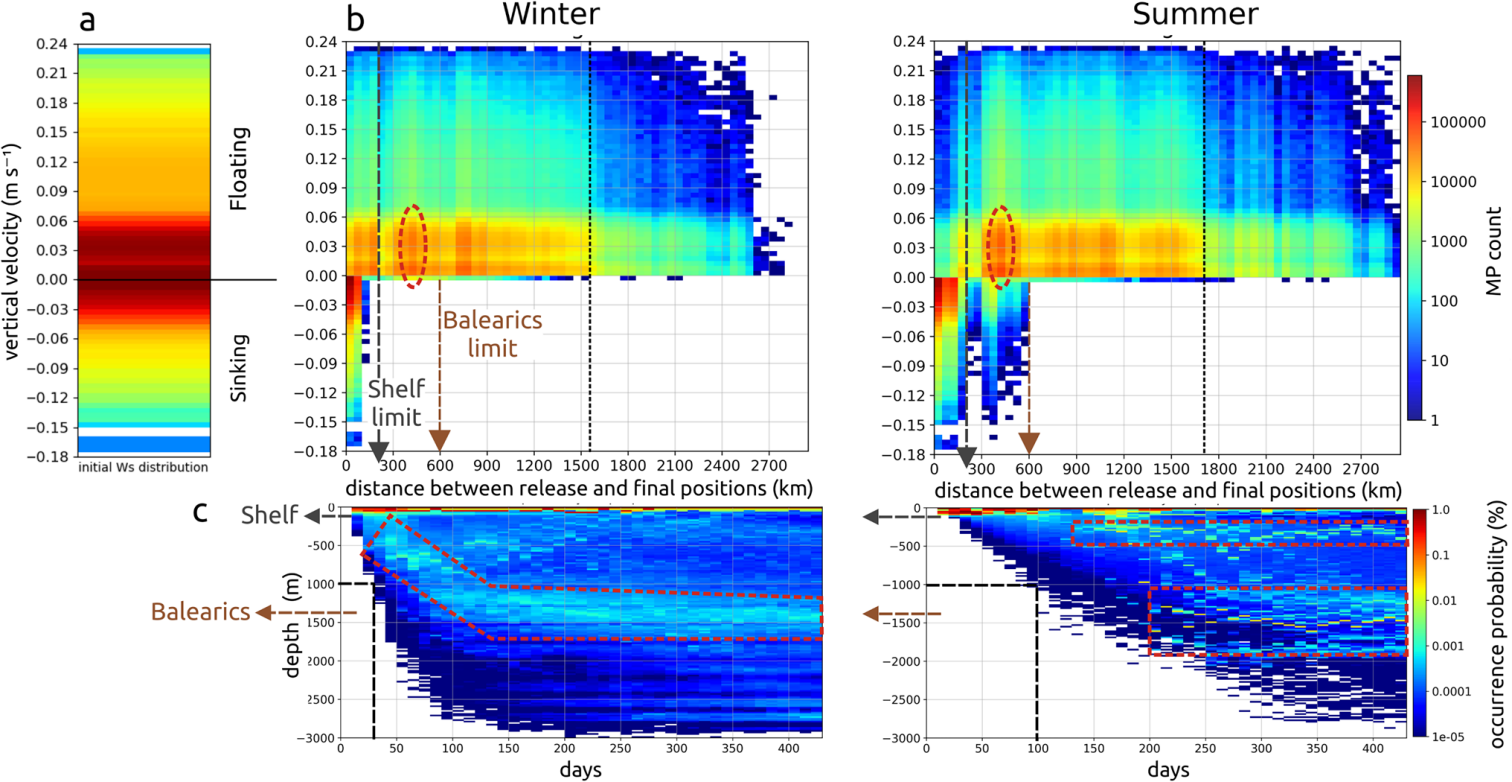
**a** Distribution of MPs released in the simulation as a function of their rising and sinking velocities, **b** straight line distance traveled by MPs from the Rhone mouth to their final position after 1 year of advection as a function of their vertical velocities, and **c** time evolution of the depths reached by MPs in the Mediterranean during 1 year of advection

Floating MPs were mostly grouped along the cyclonic side of frontal structures (in red on Fig. 5): spread in the North Balearic Front in winter (Fig. 5a) and concentrated along the Pyrenees Front in summer (Fig. 5b). The MP aggregation along fine, elongated structures between eddies showed that MPs generally disperse through filaments. In winter, the deep convection developed a sub-mesoscale soup (McWilliams 2019) inside the northwestern cyclonic circulation (Fig. 5a), away from strong currents where small current lines were abundant, more chaotic, and structures such as mesoscale eddies were less identifiable. This winter sub-mesoscale soup spread floating MPs very efficiently (Fig. 5a). In summer, because mesoscale and ephemeral sub-mesoscale structures were less developed and vorticity values were weaker than in winter, the impact of persistent frontal dynamics was more visible (Fig. 5b). The Pyrenees Front trapped floating MPs, constituting the major pathway for MP transfers. After 1 month of release, the concentration gradients between the filaments of the Pyrenees Front and their surroundings were much stronger for MPs released in summer than their equivalent in the Balearic Sea and along the North Balearic Front in winter (Fig. 5). This showed that the clustering of floating material was stronger when sub-mesoscale activity was restricted to persistent fronts.

#### Preferential trajectories

In winter and spring (Fig. 6a, b), the floating MP dispersion was much more widespread than in summer. The North Balearic Front (around 40$^{\circ }$ N) was the main barrier for southward dispersion, slowing the penetration of MPs into the Algerian basin. Contrary to its role as an effective barrier along the Gulf of Lion slope, the Northern Current in the Balearic Sea did not effectively channeled MPs towards the Strait of Ibiza. Particles were dispersed at the surface of the Balearic Sea thanks to the presence of cross-shore current branches that adjusted along the thermohaline gradients present in particular around the Gulf of Lion convection zone (see, for example, the current between Barcelona and Menorca in winter in Fig. 2e). MPs then outflowed southward between the islands and largely bypassed Menorca to the northeast. Higher MP concentrations were indeed observed at the north of the Balearic Islands compared to the more protected southern part of the islands (Ruiz-Orejón et al. 2018).

On the contrary, in late summer and autumn (Fig. 6c, d), the lower extent of MP dispersion illustrated the imprint of the Northern Current, the Pyrenees Front, and its northeastern extension towards Corsica (called the Pyrenees-Corsica front zone by Barral et al. 2021). In September, the front bounding the Northern Current along the Catalan coast led to a very concentrated MP occurrence in the northernmost part of the Balearic Sea, protecting the islands, while the recirculation associated with the Pyrenees Front channeled the MPs (around 4$^{\circ }$ E and 41.5$^{\circ }$ N) to the East, partly bypassing the Balearic Sea. Then, the eastwards recirculation split in two trajectories in the center of the basin, one towards Corsica joining the West Corsican Current and the Northern Current in the Ligurian Sea (Fig. 6d), and the other towards Sardinia and the south of the basin.

Consequently, the coastal zones of the Balearic Islands may be much more protected in summer, whereas the coasts of Sardinia, Corsica, and the Ligurian Sea were more protected in winter. The summer situation also resulted in zero MP leakage through the Strait of Ibiza protecting Spain’s southern coastal area from pollution emitted by the Rhone (Fig. 6c, d). Finally, as observed for the anticyclonic cell in the Gulf of Lion, the effect of the anticyclone present in the Algerian basin was highlighted: it showed a very low MP occurrence probability in its center whatever the season (Fig. 6a, b, d).

### Travel distances

**Fig. 8:**
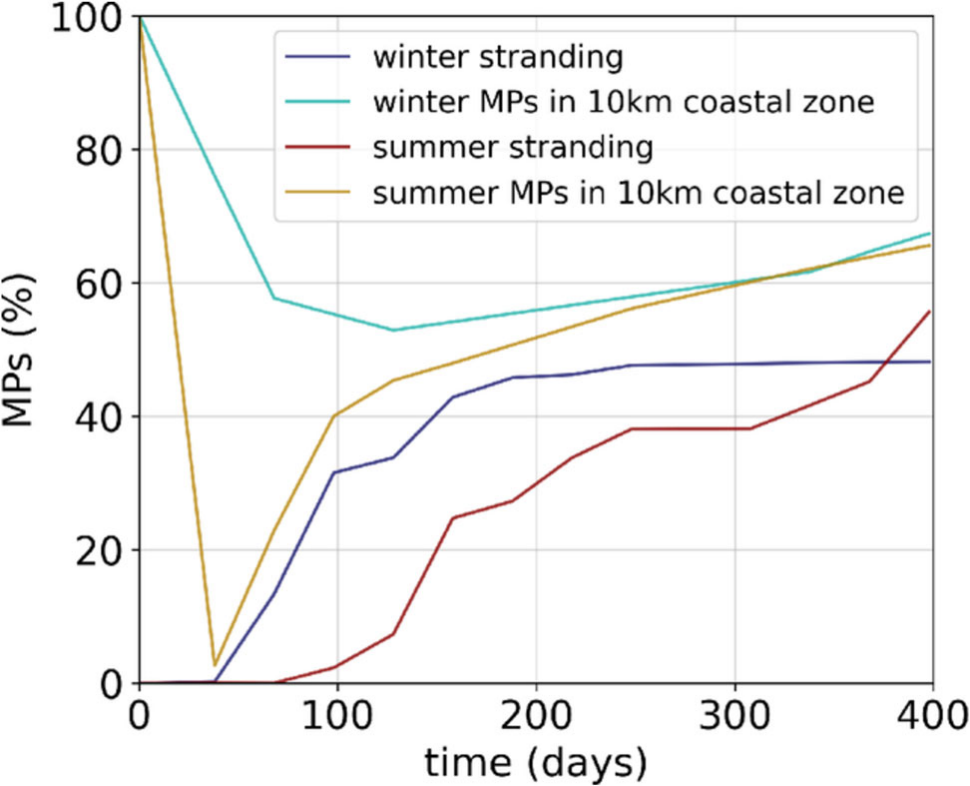
Time evolution of MP rate stranded at the coast and accumulated in the 10 km coastal zone for the whole Mediterranean. MPs were released daily during the first month of simulation, day 1 to 31, and advected for 1 year, until day 400

**Fig. 9:**
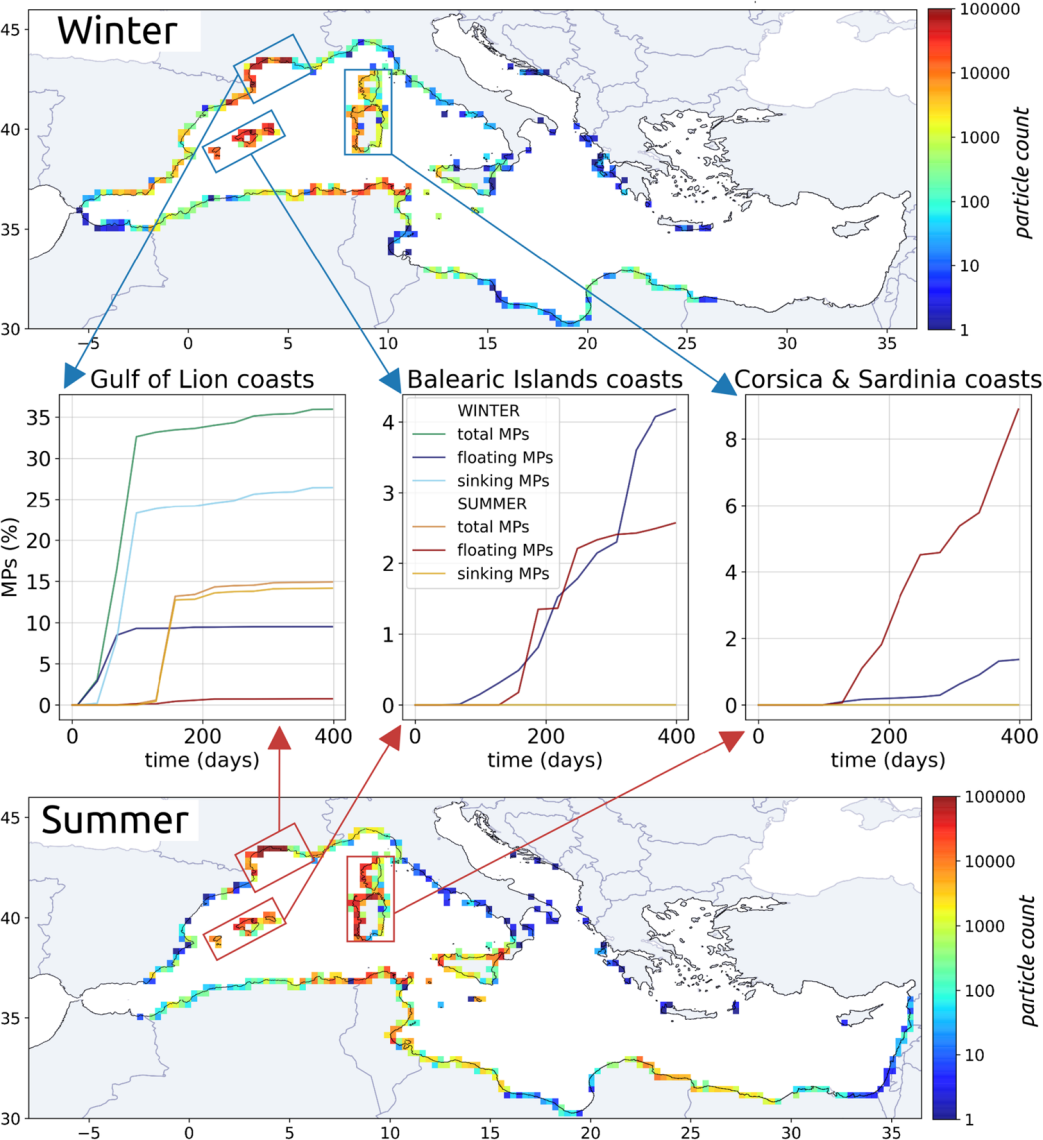
Stranding on the Mediterranean coasts after 1 year of simulation for the winter and summer scenarios. The middle plots show the temporal evolution of stranding on the coasts of the Balearic Islands, Gulf of Lion, Corsica, and Sardinia for floating and sinking MPs. Only 3 curves are shown for the islands because no sources are simulated there and no dense MPs reached their coasts

Seasonal dynamics in the Gulf of Lion and thermal fronts had an impact on the distances traveled by MPs in the surface layer and at depth. MPs released in summer traveled more slowly (Fig. 7c), but the distance between their release and final position was greater compared to winter releases (Fig. 7b), due to a favored and more direct summer export of MPs off the continental shelf rather than a stronger winter coastal stranding (Figs. 8 and 9).

The favored export to the open sea in summer was illustrated by a floating MP gap within a 300 km radius of the Rhone, while in winter, many more MPs were found until 200 km on the shelf (Fig. 7b). Then off the shelf, a homogeneous distribution of traveled distances of floating MPs was observed until 1600–1700 km, with slightly highlighted signal in the Balearic Sea around 400 km distance, along the Spanish continental slope opposite Majorca. For sinking MPs, the whole range of sinking velocities reached the Balearic’s seafloor in summer (until 600 km distance), whereas in winter, only the lightest sinking MPs were present (with the densest still trapped 100–150 km from the Rhone). Sinking MPs were found on average 12 to 15 times closer to the Rhone than floating MPs. Maximum distances traveled by floating MPs over a year were about 2800–3000 km toward the eastern basin. However, a few dozen sinking MPs, mainly fibers, were found up to 1500–1800 km maximum from the Rhone toward the Atlantic (Fig. 7b).

Although the quantity of sinking MPs exported off the continental shelf was lower in winter than in summer, Fig. 7c showed that light sinking MPs released in winter reached depths of 1000 m more quickly (after 1 month) than those released in summer (after 3–4 months), due to stronger deep winter currents. This export was then followed by a deep drift of a further 3 months to be deposited massively on depths of about 1100–1800 m corresponding to the Balearic’s seafloor (Fig. 7c). At such depths, sinking MPs did not manage to exit at the Strait of Ibiza, which is about 800 m deep, and were advected northerly by the Balearic Current along the islands slope. Thus, the seafloor of the Balearic Sea accumulated the Rhone sinking MPs, rapidly after a winter release when transit times from the Gulf of Lion were short, but more massively after a summer release when exports off the shelf were slower and more numerous. Finally, a stronger deposit occurred in summer on the shelf at depths of 300–500 m.

### Stranding at the coast

Regional approaches using kilometer resolution grid, like ours, do not allow to finely resolve coastal dynamic processes. For instance, no remobilization due to wind, waves, or tides was simulated here, and stranding can occur along any coast depending on the circulation, i.e., the diversity of coastlines is not accounted for. However, some authors suggest that remobilized plastics tend to remain mostly within the coastal zone and strand again a few km further away (Zhang 2017; Isobe et al. 2014), suggesting that virtual MP resuspension in coastal grid cells would not significantly alter the final simulated distribution of strandings. Therefore, the results described hereafter should be considered as indicators of stranding risk and not realistic quantification.

After 1 year of simulation (day 400 in Fig. 8), the stranding rates at the coast were similar in both seasonal scenarios (48% in winter and 51% in summer) resulting from an accumulation of 67% MPs in the 10 km coastal zone. However, the patterns were very different in the first months of simulation: after 3 months, 30% MPs stranded in the Gulf of Lion in winter but nearly 0 in summer (day 90 in Fig. 8). Due to the deep winter coastal currents directed towards the shore near the Rhone mouth (Fig. 4c), only 50% of the MPs left the 10 km coastal zone in winter, contrary to 98% in summer (Fig. 8).

As a result, winter stranding was much more concentrated in the vicinity of the Rhone mouth and in the bay of Fos-sur-Mer, while summer stranding was more extended from the Rhone mouth to the Cap d’Agde (maps on Fig. 9). Stranding on the western coasts of the Gulf of Lion from Cap d’Agde to Cap de Creus was less numerous in summer than in winter linked to the presence of the anticyclonic eddy in this region during summer releases (Fig. 3b, d). Stranding rates on the Gulf of Lion coasts (left plot on Fig. 9) in winter (35%) were twice that of summer (15%). Stranding of sinking MPs occurred only in the Gulf of Lion because once exported to the open sea, they reached greater depths that did not allow them to return on the continental shelves. Stranding of floating MPs in the Gulf of Lion coasts was lower: 10% in winter and only 1.5% in summer (left plot on Fig. 9).

Stranding of floating MPs exported by the Rhone was distributed over the entire Mediterranean coastline. Hotspots were observed along the northern coasts of the Balearic Islands, the western coasts of Corsica and Sardinia, and the northeastern Algerian and northern Tunisian coasts (maps on Fig. 9). Major differences between the winter and summer scenarios were particularly visible for the Balearic Islands and for Corsica and Sardinia (middle and right plots on Fig. 9). After 1 year, only 1.5% of total MP inputs released in winter stranded on the coasts of Corsica and Sardinia (2.3% of floating MPs), while the stranding rates of MPs released in summer were multiplied by 6 reaching 9% of total inputs (13% of floating MPs). Although the difference was less marked, the trend was reversed for stranding on the Balearic coasts: the MPs released in winter were almost twice as numerous as those released in summer: 4.2% of total inputs in the winter scenario and 2.5% in the summer scenario (6.6% and 4.5% of floating inputs).

### Annual MP budget from the Rhone River to the Mediterranean Basin

**Fig. 10:**
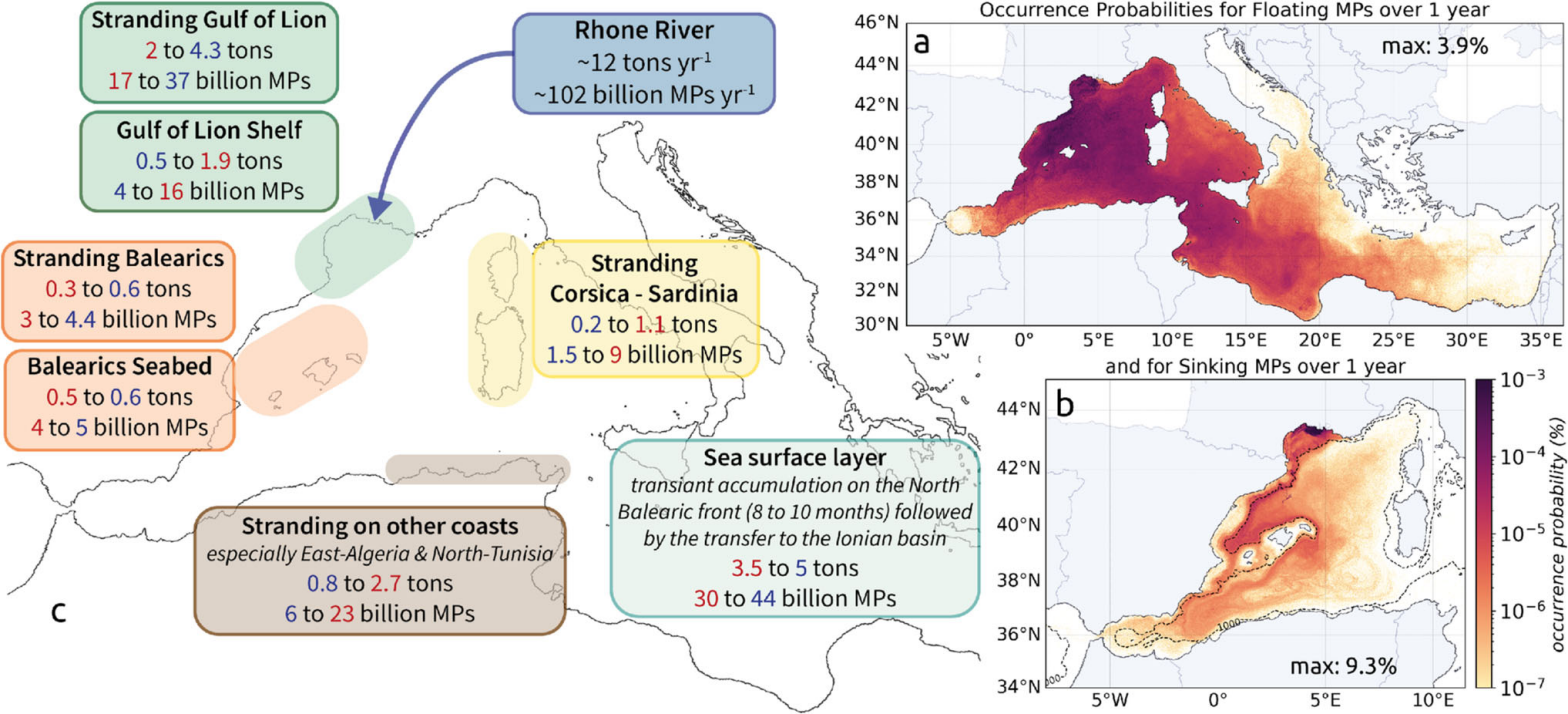
Annual occurrence probabilities of **a** floating MPs, **b** sinking MPs, and **c** Rhone annual fluxes budget, numbers in blue are based on the winter scenario and in red on the summer scenario to provide minimum and maximum estimates

The two seasonal scenarios were combined by extrapolating the summer situation from August to October (during the Pyrenees Front period) and the winter situation for the remaining months to estimate occurrence probabilities on a 1-year scale (Fig. 10a, b). An estimate of the annual budget for transfers from the Rhone to the most impacted regions has been deduced from this (Fig. 10c).

#### Sea surface transfers of MPs

Despite the strong temporal variability, generally confirmed by observations (Schmidt et al. 2018; Lefebvre et al. 2019), the Gulf and the Balearic Sea exhibited the maximum occurrences of floating MPs over a year. As they were not permanent, eddies did not trap MPs over long periods. Throughout the year, the MP concentrations tended to homogenize over the whole western basin as well as towards the eastern basin for long-life MPs (Fig. 10a). Some floating MPs from the Rhone River reached the eastern basin through the Strait of Sicily after 2 to 3 months of advection. This transfer was quicker for summer-released MPs than winter ones. After 7 months of advection, some floating MPs released in summer had already reached the Levantine Basin while winter MPs had just dispersed over the Ionian Sea, due to a longer retention by seasonal fronts in the western basin. It confirmed the high dissipative character of the Northwestern and Algerian basins, observed by Weiss et al. (2024). The annual budget showed that 50 to 60% floating MPs discharged by the Rhone were exported to the Algerian Basin before being further transferred to the Eastern Mediterranean which led to high concentrations in the Ionian Sea. Thus, the Rhone MPs reached almost the entire Mediterranean basin over 1 year (except the Aegean Sea, the Northern Adriatic, and the Northeast of the Levantine basin). A total amount of 3.5 to 5 t, i.e., 30 to 44 billion floating MPs, was still drifting in the surface layer (Fig. 10c).

#### Seafloor MP hotspots

High retention of sinking MPs occurred in the vicinity of the Rhone, mainly westward of the mouth and on the shelf (Fig. 10b). This was consistent with the high abundance of plastics on the seafloor from visual and bottom trawl surveys in the Gulf of Lion (Galgani et al. 1995; Spedicato et al. 2019). Sinking MPs slowly exited the shelf at the southwest and were channeled by the Northern Current towards the Balearic seafloor, highlighted as a preferred destination for sinking MPs, with an estimated budget of 0.5 to 0.6 $t \ yr^{-1}$ (Fig. 10c). At depth, the seasonality had little influence on sinking MP pathways, but had a notable impact on the quantities dispersed. After a year, sinking MPs just reached the Algerian Basin and the Alboran Sea due to their much slower deep dispersion (Fig. 10b). Only about 100 MPs (0.001 to 0.002% of the Rhone inputs) reached the Atlantic Ocean via the Strait of Gibraltar.

#### Coastal contamination by MPs

Our annual budget suggests that 20 to 50% of the MPs emitted by the Rhone remained in the coastal system close to the source, through massive stranding and deposit on the shelf. This retention was strongly accentuated in winter, when most of the river MPs were actually discharged due to higher water discharges. Winter current conditions and strong south-southeasterly winds (such as in March 2013) led to twice the amount of summer stranding on the coasts of the Gulf of Lion (4.3 vs 2 $t \ yr^{-1}$ of MPs, i.e., 36 vs 16% of the total annual inputs; Fig. 10c). This identified the Gulf of Lion as a plastic pollution hotspot. It is consistent with previous modeling studies that showed that most of the dense MPs reached the bottom very close to their sources (Soto-Navarro et al. 2020), making the coastal zone an important accumulation zone. Areas of high deposition have been identified such as estuaries (Defontaine et al. 2020; Peng et al. 2017), pro-deltas (Pellegrini et al. 2023), the floor in front of river mouths (Acha et al. 2003; Galgani et al. 2000), and beaches adjacent to river mouths (Atwood et al. 2019; Constant et al. 2020; Rech et al. 2014). Observation campaigns along the coast could be used to verify this in certain areas targeted by the model.

In late summer to autumn, the preferential MP transfers along the Pyrenees Front redirected the floating MPs further east of the basin, protecting the coasts of the Balearic Islands with 0.3 $t \ yr^{-1}$ MPs stranded instead of 0.6 after the winter release (Fig. 10c). It led to increase stranding along the western coasts of Corsica and Sardinia Islands with 1.1 $t \ yr^{-1}$ instead of 0.2 after winter release (Fig. 10c), as well as to higher floating MP concentrations in the Ligurian and Tyrrhenian Sea. The MP transfers from the Western to the Eastern Basin also strongly impacted the southern Mediterranean coasts, with 0.8 to 2.7 $t \ yr^{-1}$ accumulated on the Algerian and Tunisian, but also along the Sicilian and Egyptian coasts.

## Conclusion

The Mediterranean Sea has been described as one of the most polluted seas on Earth. Our study allowed a better understanding of the fate of MPs discharged by the Rhone River, one of the major contributors of MP pollution to the Mediterranean basin.

After 1 year, more than 60% of the released MPs ended up in the 10 km coastal zone, of which 50% were stranded. This suggests a rather small residence time of Rhone MPs in the Mediterranean sea. We observed that the seasonality of the Rhone freshwater discharge, together with the stratification and the high variability of wind-driven currents in the Gulf of Lion, had a strong impact on the dispersion of the MPs on the shelf, leading to high variability in accumulation zones. Stranding was especially important in the Gulf of Lion, mainly in winter being 2 times higher than in summer for sinking MPs and 6 times higher for floating MPs. The reduced stranding in summer led to higher exports and greater distances between the initial and final positions than after the winter release. However, after 1 year, 40% (winter)–33% (summer) of the MPs released by the Rhone still remained in the Gulf of Lion, which suggests that a significant part of the MP pollution observed in this area is originating from local sources and not advected from remote pollution hotspots. This type of information is crucial in order to convince local stakeholders to fight more efficiently plastic pollution prior to its introduction in the marine environment.

Off the shelf, the frontal dynamics was conducive to the formation of sub-mesoscale eddies and filaments that resulted in aggregation and preferential transfers of floating MPs. The clustering of floating material was stronger when sub-mesoscale activity was limited to persistent fronts such as the Pyrenees Front in summer. In winter, strong mixing created more sub-mesoscale that resulted in particle advection over a wider area when the frontal interface corresponded to the North Balearic Front. The Pyrenees Front channeled floating MPs in summer and fall toward Corsica and Sardinia, protecting the Balearic Islands, while in winter and spring, MPs were preferentially spread by eddies and filaments in the Balearic Sea. When they are formed, anticyclonic cells protected areas such as the western Gulf of Lion in summer or the Algerian Basin throughout the year, with high temporal and spatial variability in these structures.

For further research, better understanding of the annual variability of MP river discharges, the effect of heavy rainfall or flooding, and trapping processes as stranding or sedimentation is needed to improve our ability to develop new insights on plastics cycle modeling in the marine environment and to predict more accurately the resulting mass budgets and accumulation zones in the oceans.

## Data Availability

The model configuration and the simulations can be shared on request.

## Code Availability

Code SYMPHONIE available from INSU-certified service SIROCCO (sirocco.obs-mip.fr).
